# Feasibility of a physical activity intervention during and shortly after chemotherapy for testicular cancer

**DOI:** 10.1186/s13104-017-2531-y

**Published:** 2017-06-15

**Authors:** Lene Thorsen, Camilla Kirkegaard, Jon Håvard Loge, Cecilie E. Kiserud, Merethe Lia Johansen, Gunhild M. Gjerset, Elisabeth Edvardsen, Hanne Hamre, Tone Ikdahl, Sophie D. Fosså

**Affiliations:** 1 National Advisory Unit on Late Effects After Cancer Treatment, Oslo University Hospital, Box 4950, Nydalen, 0424 OSLO Oslo, Norway; 2 Clinical Manager, Medical Affairs, Coloplast A/S, Humlebæk, Denmark; 3 Department of Behavioral Sciences in Medicine, University of Oslo/Regional Advisory Unit in Palliative Care, Oslo University Hospital, Oslo, Norway; 4 Department of Cancer Rehabilitation, Oslo University Hospital, Oslo, Norway; 5 Department of Pulmonary Medicine, Oslo University Hospital, Oslo, Norway; 6 Department of Oncology, Akershus University Hospital, Lorenskog, Norway; 7 Akershus University Hospital, Lorenskog, Norway

**Keywords:** Testicular cancer, Chemotherapy, Physical activity, Feasibility

## Abstract

**Background:**

Given the risk of developing acute and long-term adverse effects in patients receiving cisplatin-based chemotherapy for testicular cancer (TC), risk-reducing interventions, such as physical activity (PA), may be relevant. Limited knowledge is available on the challenges met when conducting PA intervention trials in patients with TC during and shortly after chemotherapy. The aims of the present feasibility study are therefore to determine patient recruitment, compliance and adherence to a PA intervention.

**Results:**

Patients with metastatic TC referred to cisplatin-based chemotherapy were eligible. They followed an individual low-threshold PA intervention, including counseling from a personal coach during and 3 months after chemotherapy. Outcomes were recruitment rate, compliance rate and adherence to the intervention including preferences for type of PA and barriers for PA. During 8 months 12 of 18 eligible patients were invited, all consented, but three dropped out. Walking and low intensity activities were preferred and nausea and feeling unwell were the most often reported barriers towards PA.

**Discussion:**

In order to achieve adequate recruitment, compliance and complete data in future PA intervention trials, close cooperation with treating physicians, individual PA plans and availability of personalized coaching are required.

*Trial registration* NCT01749774, November 2012, ClinicalTrials.gov

**Electronic supplementary material:**

The online version of this article (doi:10.1186/s13104-017-2531-y) contains supplementary material, which is available to authorized users.

## Background

Testicular cancer (TC) is the most common cancer among men between 18–40 years of age [1]. Standard treatment for patients with low- or intermediate risk metastatic disease is chemotherapy including three or four cycles with etoposide and cisplatin with or without bleomycin (EP or BEP) [2]. This treatment often leads to acute toxic effects such as bone marrow suppression, nausea, thromboembolic events, reduced kidney function, and peripheral neuropathy, resulting in reduced well-being and physical capacity during treatment [3]. After chemotherapy, long-term TC survivors are at risk of several late effects and display a higher risk of diabetes and myocardial infarction compared to age-matched men from the general population [4, 5]. Interventions that can reduce the risk of acute and long-term adverse effects are therefore warranted.

Several meta-analyses have concluded that physical exercise is a feasible and beneficial intervention for several health outcomes in cancer patients, however most studies have been conducted in breast cancer patients and after cancer treatment [6–9]. In a recent review, only 17 studies were identified assessing the effects of physical exercise during adjuvant cancer treatment, of which 14 studies included breast cancer patients, two studies lung cancer patients and one study patients with several diagnoses [10]. Studies examining the feasibility and effects of physical activity (PA) during chemotherapy treatment in patients with other diagnoses than breast cancer, such as cisplatin-based chemotherapy in patients with metastatic TC are needed [11].

One rationale for initiating PA during chemotherapy is to maintain physical fitness, both in terms of aerobic capacity and muscle strength, and thereby possibly reduce troublesome side-effects and increase well-being. On the other side acute side-effects might be barriers towards PA, and more knowledge on the optimal content of a PA intervention during such treatment and how these interventions should be conducted in practice is needed. Before planning and implementing randomized clinical trials (RCTs) including PA in patients with metastatic TC during chemotherapy, patient recruitment, compliance and patients’ adherence to such intervention are important to ensure that a RCT can be successfully performed. A review by Oldervoll et al. [12] focused on recruitment, compliance and adherence to PA interventions during and after cancer treatment. In 12 RCTs, the average recruitment rate was 43%, the average compliance rate was 86% and the adherence to the interventions ranged from 72–100%. The majority of the participants (62%) were breast cancer patients during and after chemotherapy [12].

The young age, male gender and the BEP/EP regimen, with its specific toxic effects, might interfere differently on the feasibility in PA studies in patients with metastatic TC than other cancer populations. Therefore, the aims of the present feasibility study are to determine patient recruitment, compliance and adherence to a PA intervention during and shortly after cisplatin-based chemotherapy, in patients with newly diagnosed metastatic TC.

## Methods

### Patients and treatment

This feasibility study was performed as a single institutional prospective one-armed intervention study at Oslo University Hospital (OUH) from December 2012 to July 2013 (8 months). OUH is the only institution treating patients with newly diagnosed TC within a population of approximately 2.8 million individuals, living in the south eastern part of Norway. Most patients undergo orchiectomy at their local hospital, but are thereafter referred to OUH for staging and eventually further treatment. Risk-adapted treatment of patients with metastatic TC comprises three or four cycles of BEP or four EP [2]. Each cycle of 21 days includes 5 days at OUH receiving chemotherapy infusions (days 1–5), and 16 days at home (days 6–21). At day 15, the patients receive bleomycin intravenously at their local hospital. Post-chemotherapy residual tumor often requires surgery, most frequently retroperitoneal lymph node dissection.

Patients eligible for the present study fulfilled the following criteria: newly diagnosed metastatic TC, planned for three or four cycles with BEP/EP, without physical and psychological co-morbidities contraindicating the assessments and/or intervention as assessed by the responsible oncologist, age ≥18 years and no prior cancer (except non-melanoma skin cancer).

### Intervention

Due to lack of literature regarding PA in patients with metastatic TC, we conducted upfront semi-structured interviews among 11 men who previously had received BEP/EP chemotherapy for TC. These interviews revealed reduced wellbeing, fatigue and weakness especially on the days 4–7 in each cycle. Wellbeing gradually decreased throughout the treatment period. After completion of chemotherapy, the interviewed patients reported that recovery to pre-treatment physical capacity could take several months. Most interviewed patients expressed skepticism towards performing high intensity (HI) physical exercise during chemotherapy and preferred PAs with low intensity (LI) or moderate intensity (MI).

**Fig. 1:**
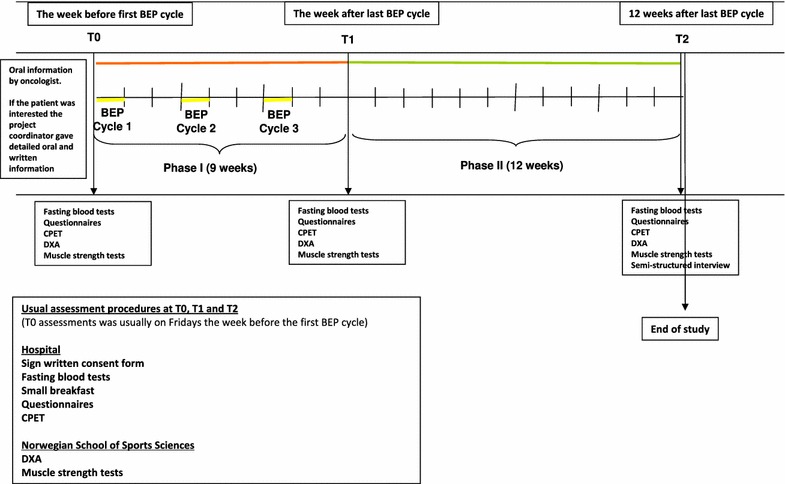
Design of the feasibility study and timeline for each patient

On this background the intervention in the present feasibility study was designed as an individual low-threshold PA intervention during (Phase I) and shortly after (Phase II) chemotherapy (Fig. 1). The intervention aimed to avoid inactivity and maintenance of physical capacity. All patients got a personal coach to motivate, counsel and encourage them to follow the public health PA guidelines (≥150 min MI activity per week or ≥75 min HI activity) [13]. During the first week of treatment an individualized PA plan was developed. The patient could choose different types of PAs and the intensity should be adapted to the patient’s day-to-day wellbeing. The coach contacted the patient in person at least once during each hospitalization, and if desirable, counseled the patient during PA sessions at the training centre at the hospital. Between the hospitalizations the coach phoned the patient at least once per week, but no face-to-face counseled PA sessions were performed outside the hospital.

Phase II started after the last BEP/EP cycle and lasted for 12 weeks. The coach and the patient worked out an individual PA plan depending on the patient’s preferences. All PAs were to be performed near by the patients’ home. The coach phoned the patient every second week, encouraging him to increase the frequency, duration and intensity of PAs, at least to a level corresponding to the public health PA guidelines.

### Feasibility outcomes

#### Patient recruitment, refusal and compliance

Eligible patients were recruited by the responsible oncologists 1–2 weeks before the first BEP/EP cycle (Fig. 1). After short oral information by the treating oncologist, the patients received written information and more detailed oral information from the study coordinator if they expressed an interest in participating. The *recruitment rate* was defined as the proportion of invited patients among eligible patients. Reasons for eligible patients not being invited were identified retrospectively. *Refusal rate* was defined as the proportion of patients among the invited that did not consent to participate. *Completion rate* was defined as the proportion of consenting patients who completed any measurement at the final assessment.

The patients were evaluated during the week before the first BEP/EP cycle (T0), the week after the last BEP/EP cycle (after phase I) (T1) and 12 weeks after the last BEP/EP cycle (after Phase II) (T2) (Fig. 1). The assessments included fasting blood sampling, questionnaires, a cardiopulmonary exercise test (CPET), dual-energy X-ray absorptiometry (DXA) and muscle strength tests. *Assessment completion rate* was defined as the proportion of completed assessments, at T0, T1 and T2 separately and summed up.

#### Adherence to the intervention

The patients were asked to report all types of PA including the intensity and duration in a PA log during each chemotherapy cycle. Intensity was reported according to the Borg scale [14]. PAs rated below 12 at the Borg scale were categorized as LI sessions, activities rated from 12 to 14 were categorized as MI sessions, and activities above 14 as HI sessions [14].

At T2 the patients went through a semi-structured interview, including questions related to PA preferences (Table 4) and barriers asking; “On a scale from 0 to10, how did the listed adverse effects (Table 5) hamper your level of PA during chemotherapy?” (1 = not all to 10 = to a very high degree).

### Ethics

The study was approved by the Regional Committee for Medical and Health Research Ethics, South-East Region (2011/2008/REK South-East A) and registered in ClinicalTrial.gov (NCT01749774). Eligible patients willing to participate signed an informed consent form prior to testing at T0.

## Results

### Patient recruitment, refusal and compliance

**Fig. 2:**
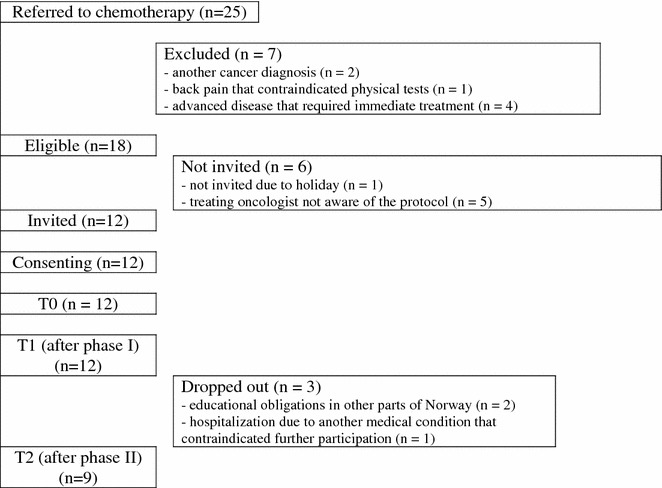
Patient flow through the study

From December 2012 to July 2013, 18 of 25 patients with stage II–IV TC were eligible for the study, of which 12 patients (mean age 36 years) were invited, all of them receiving three BEP cycles (Fig. 2; Table 1).

**Table 1:** Baseline characteristics of included patients (n = 12)

	Mean (SD)
Years of age	36 (7.7)

	N
Married/cohabitant	9
Education	
Primary/secondary school	10
College/university	2
Histology	
Seminoma	7
Non-seminoma	5
Stage^a^	
IIa/b	10
IIIa/b^b^	2
Treatment	
3BEP	12

^a^According to Royal Marsden Hospital Stage system [15]

^b^Patients with stage IIIa/b had recidiv from their seminoma

The study achieved a recruitment rate of 66%, logistics were the main reason participants did not enter the study, particularly summer holiday and oncologists not being aware of the study. None of the invited patients refused to participate (refusal rate: 0%). No patients dropped out between T0 and T1, but three patients dropped out after T1 (but before phase II of the intervention), because of educational obligations outside the region (n = 2) and hospitalization due to a medical condition (n = 1), giving a compliance rate of 75%.

At T0, T1 and T2 the assessment completion rates were 92, 89 and 62%, respectively. Of 216 possible assessments 175 (81%) were completed. The lowest completion rate was observed for DXA-scans, due to lack of staff at the lab (Table 2).

**Table 2:** Assessment completion among consenting patients (n = 12)

	T0	T1	T2	Total
Questionnaire	12	12	9	33
CPET	11	11	8	30
DXA	9	9	7	25
Leg press	10	10	7	27
Chest press	12	11	6	29
Pull down	12	11	8	31
Total possible assessments	72	72	72	216
Total completed assessments	66	64	45	175
Assessment completion rate (%)	92	89	62	81

Reasons for missing data: CPET: T0, 1 due to lack of personnel at the lab; T1, 1 due to lack of personnel at the lab; T2, 1 due to lack of personnel at the lab. DXA: T0, 3 due to lack of personnel at the lab; T1, 3 due to lack of personnel at the lab; T2, 2 due to lack of personnel at the lab. Leg press: T0, 1 due to pain in the back and 1 due to surgery; T1, 1 due to pain in the back and 1 due to thrombosis in the leg; T2, 1 due to pain in the back and 1 due to thrombosis in the leg. Chest press: T1, 1 due to pain in the arm; T2, 1 due to pain in the arm and 2 due to broken chest press instrument. Pull down: T1, 1 due to pain in the arm; T2, 1 due to pain in the arm

### Adherence to the intervention

Eleven patients completed their PA log during chemotherapy (Table 3). During the first cycle (week 1–3), three patients met the PA guidelines each week (only one patient all 3 weeks). During the second cycle (week 4–6), two patients met the PA guidelines in week 4 and 6, whereas three patients met the PA guidelines in week 5. During the third cycle (week 7–9) one patient met the PA guidelines each week (different patient each week) (Table 3) and (Additional file 1).

**Table 3:** Physical activity log during each BEP cycle (n = 11)

	N	HI sessions (≥15 BS)	MI sessions (12–14 BS)	LI sessions (≤11 BS)
BEP 1				
Week 1				
Meeting guidelines (n)	3			
No activities reported (n)	2			
Patients (n)/no of sessions performed		3/9	6/9	6/25
Mean duration of the sessions [min. (range)]		47 (15–120)	35 (15–60)	36 (15–90)
Types of activities		ST, bicycling, running	Bicycling, ST, running, walking, SAG	Walking, HK, SAG, jogging
Week 2				
Meeting guidelines (n)	3			
No activities reported (n)	0			
Patients (n)/no of sessions performed		5/10	6/14	9/37
Mean duration of the sessions [min. (range)]		46 (25–120)	39 (20–90)	28 (10–95)
Types of activities		TT, CCS, bicycling, walking, ST	TT, walking, bicycling, ST, Football, SAG	Walking, ST
Week 3				
Meeting guidelines (n)	3			
No activities reported (n)	2			
Patients (n)/no of sessions performed		6/9	5/10	6/27
Mean duration of the sessions [min. (range)]		43 (20–90)	42 (20–90)	25 (10–68)
Types of activities		CCS, ST, running, TT, jogging	TT, CCS, walking, ST, jogging, SAG	Walking, ST, gardening
BEP 2				
Week 4				
Meeting guidelines (n)	2			
No activities reported (n)	3			
Patients (n)/no of sessions performed		3/6	2/2	6/23
Mean duration of the sessions [min. (range)]		87 (20–160)	58 (40–75)	40 (10–240)
Types of activities		Jogging, CCS, walking, ST	Walking, SAG	Walking
Week 5				
Meeting guidelines (n)	3			
No activities reported (n)	2			
Patients (n)/no of sessions performed		2/4	4/5	6/19
Mean duration of the sessions [min. (range)]		36 (10–60)	141(30–360)	33 (10–60)
Types of activities		CCS, walking	Curling, hiking, HK, ST, walking	Walking, gardening, swimming, HK, SAG
Week 6				
Meeting guidelines (n)	2			
No activities reported (n)	3			
Patients (n)/no of sessions performed		3/4	3/6	5/31
Mean duration of the sessions [min. (range)]		28 (10–58)	94 (30–270)	41 (20–240)
Types of activities		CCS, walking, cross-fit	Paddling, football, SAG, walking	Walking, SAG, gardening, HK, SAG
BEP 3				
Week 7				
Meeting guidelines (n)	1			
No activities reported (n)	5			
Patients (n)/no of sessions performed		2/4	0	4/10
Mean duration of the sessions [min. (range)]		74 (30–180)		27 (10–45)
Types of activities		Running, walking		Walking, gardening
Week 8				
Meeting guidelines (n)	1			
No activities reported (n)	7			
Patients (n)/no of sessions performed		1/1	2/4	1/2
Mean duration of the sessions [min. (range)]		30	81 (40–160)	75 (60–90)
Types of activities		ST	Walking, ST, SAG	Gardening, SAG
Week 9				
Meeting guidelines (n)	1			
No activities reported (n)	6			
Patients (n)/no of sessions performed		1/1	2/6	1/2
Mean duration of the sessions [min. (range)]		20	43 (20–90)	120 (120)
Types of activities		ST	Walking, SAG	Gardening

HI, high intensity; BS, Borg Scale; MI, moderate intensity; LI, low intensity; ST, strength training; SAG, session at the gym; HK, house keeping; CCS, cross-country skiing; TT, table tennis

During nine weeks of chemotherapy the number of HI, MI and LI sessions decreased gradually paralleled by an increasing proportion of patients reporting no PA at all (Table 3).

Nine of 12 underwent a semi-structured interview on their preferences and experienced barriers related to PA at T2. Walking and LI activities were most preferred (Table 4). The most often reported barriers towards PA during chemotherapy were nausea, feeling unwell, reduced general condition and being exhausted/tired (Table 5). Eight patients said that they preferred a flexible program and five would have preferred a personal trainer during the exercise sessions. Six patients said that they could have been more active than they actually had been and four said they could have been pushed more by their personal coach.

**Table 4:** Physical activity preferences during chemotherapy (n = 9)

	N
What kind of activity did you prefer during chemotherapy (open question)	
Walking	6
Walking/jogging/stair climbing	1
Outdoor activities	1
Pleasurable activities	1
Which intensity did you prefer?	
Low	5
Low/moderate	2
Moderate/hard	1
Adjusted to the condition	1
What do you think is most suitable during BEP treatment?	
A strict exercise program	1
A flexible program	8
Would you have preferred a personal trainer during the PA sessions outside the hospital?	
Yes	5
No	4
Would you have been able to be more active than you actually were?	
Yes	6
No	3
Could your coach have pushed you more?	
Yes	4
No	5

**Table 5:** Physical activity barriers during chemotherapy (n = 9)

	Mean value
Nausea	5.7
Feeling unwell	5.7
Reduced general condition	4.8
Exhausted/tired	4.6
Headache	3.6
Breathlessness	3.6
Diarrhea	2.6
Reduced muscle strength	2.6
Dyspnea	2.4
Constipation	2.1
Increased heart rate	1.6
Neuropathy	1.4

“On a scale from 0 to 10, how did the following adverse effects hampered your level of physical activity during chemotherapy?” (1 = not at all to 10 = to a very high degree). Mean value for the nine patients that were interviewed, for each adverse effect

## Discussion

The present feasibility study illustrates several factors that need to be considered before conducting PA intervention trials in patients with metastatic TC; possible loss of eligible patients, logistic challenges related to assessments and need for face-to-face PA counseling to increase the adherence to the intervention. On the other hand, the interest to participate in PA intervention studies seems to be high among these patients during and shortly after chemotherapy.

Among all the patients referred to three or four BEP/EP during the study period, more than one fourth (7 of 25) was excluded. Four of seven patients (57%) were ineligible due to their need for immediate treatment. In future PA intervention trials this type of ineligibility should be taken into account when estimating the number of patients to be available.

Even in large cancer centres the number of patients refereed to chemotherapy for metastatic TC is relatively low, and performing multi-centre studies in future trials will reduce the recruitment period. Optimal patient recruitment also requires a well established cooperation between the involved researchers and clinicians. Our feasibility study was lead by researchers within the field of PA, and highly involved clinicians are also necessary to increase the recruitment rate in future PA intervention trials. Two patients withdrew during follow-up due to studies at universities in other parts of the country, and this is probably a more common reason for drop-out in a young cancer population than in older patients.

To examine the effects of PA interventions, comprehensive and long-lasting pre-post intervention assessments involving staff from several labs are often required. The interval from detection of the metastasis to initiation of chemotherapy is only a few days. A challenge is therefore to coordinate the assessments at the labs within the same day, preferable within half a day. Close cooperation between the researchers and the labs is required. Warning the test-leaders and allocating time for testing, with the risk that it might be cancelled at last minute might be necessary. A possible solution might be to start the first BEP/EP cycle on Monday afternoon giving the time before lunch for testing. In a hectic daily hospital life adjustments related to such a study might claim extra work for the doctors and nurses and their goodwill and cooperation is needed. The number of necessary assessments before and during the study should carefully be considered in future intervention trials.

All patients invited to participate in the study agreed. In the study of Christensen et al. [16] 73% eligible patients were willing to participate and included. The reasons for refusal were lack of time or interest or long traveling distance. The flexible and individually adjusted intervention in our feasibility study might have favored a positive attitude towards participation and been an important reason for no drop-outs during chemotherapy. On the other hand the flexible individualized approach and lack of standardized training dose for each patients, limit the possibility to conclude on the exact dose of PA that are feasible and necessary to maintain physical capacity in future intervention trials. Standardized supervised exercise sessions at the hospital might increase the effects of a PA intervention, but the inconvenience with traveling to the hospital several times per week might be a threat to the overall adherence to the intervention and external validity.

To avoid a high refusal rate in future PA intervention trials in patients with TC, we suggest that the intervention, when the patients are not hospitalized, should be conducted at fitness centres near the patients’ homes under supervision of qualified personal trainers or physiotherapists. PA near by the patients’ home might be of special importance in rural areas, and makes it easier to continue the program after the intervention period. It has been shown that patients with lung cancer performing PA after surgery had positive effects on cardiorespiratory fitness, muscle strength, and quality of life of a supervised aerobic and strength training program, supported by a personal trainer at a fitness centre near the patients’ home [17]. In the present feasibility study, the intervention was individualised and flexible according to the patient’s daily well-being, with regular counselling by the coach. Nevertheless, the level of PA during chemotherapy was lower than expected and only a few patients met the PA guidelines. Treatment related adverse effects have shown to account for more than 50% of the barriers to perform PA in cancer patients treated with chemotherapy [18]. The patients in our study reported well-known chemotherapy-related acute adverse effects as barriers to perform PA. Interestingly, after the intervention, more than half of the patients indicated that they could have been able to be more active than they actually were, and that they could have been pushed more by the coach. Christensen et al. showed that patients with TC in average performed 70% of the planned resistance training sessions during BEP supervised by a trainer. In further intervention trials more frequent face-to-face counseling by a personal trainer during the exercise sessions might be necessary to increase the PA level during BEP/EP.

## Conclusion

Several aspects should be addressed before planning and implementing PA intervention trials in patients with metastatic TC during chemotherapy. A close cooperation between researchers, the clinicians’ and staff at the labs is important in order to increase the recruitment- and assessment compliance rate. The experiences from this feasibility study suggest that the interest for PA during chemotherapy in patients with metastatic TC is high, but face-to-face counselling by a personal trainer or physiotherapist might be important to increase the level of PA during chemotherapy. The challenges regarding logistics related to inclusion and assessments procedures should seriously be taken into account when planning and performing PA intervention trial in patients with metastatic TC.

## Additional file

**Additional file 1.** Physical activity log during each BEP cycle.

## Abbreviations

TC: testicular cancer
PA: physical activity
EP: etoposide and cisplatin
BEP: etoposide, cisplatin and bleomycin
RCT: randomized clinical trials
OUH: Oslo University Hospital
HI: high intensity
LI: low intensity
MI: moderate intensity
T0: the week before the first BEP/EP cycle
T1: the week after the last BEP/EP cycle
T2: 12 weeks after the last BEP/EP cycle
CPET: cardiopulmonary exercise test
DXA: dual-energy X-ray absorptiometry
