# Supplementary material for: Feasibility of a physical activity intervention during and shortly after chemotherapy for testicular cancer
Source: BMC Res Notes. 2017 Jun 15;10:214. doi: 10.1186/s13104-017-2531-y (PMC5472911; doi:10.1186/s13104-017-2531-y)
Supplement: Supplementary file 1 — Additional file 1. Physical activity log during each BEP cycle. [file 13104_2017_2531_MOESM1_ESM.doc]

**Additional file: Physical activity log during each BEP cycle**

| **BEP 1** | **Type of activities** | **High intensity (> 15 Borg scale)** | **Moderate intensity (12-14 Borg scale)** | | **Low intensity (< 11 Borg scale)** |
| --- | --- | --- | --- | --- | --- |
| 1 | None activities (P-5 and 8)  Walking (P-1, 2, 4, 6, 9, 12 and 13)  ST (P-1, 2, 6, 7 and 10)  Cycling (P-1, 2 and 7)  Running/jogging (P-2, 7 and 13)  Housekeeping (P-2)  Activities at the gym (P-12)  **Meeting guidelines (P-1, 2, 7)** | **P-1***  2 ST sessions (30 and 120 min)  **P-2 ***  1 cycling session (50 min)  **P-7***  3 cycling/running sessions (3x60 min)  3 ST sessions (3x15 min) | **P-1***  1 cycle session (30 min)  **P-2***  1 ST session (36 min)  1 running session (30 min)  1 walking session (40 min)  **P-6**  1 ST session (20 min)  **P-9**  1 walking session (15 min)  **P-10**  1 ST session (20 min)  **P-12**  1 walking session (60 min)  1 session at the gym (60 min) | | **P-1**  4 walking sessions (4x15 min)  **P-2**  10 walking sessions (4x15, 20, 30, 52, 46 and 60x2min)  1 house keeping session (30 min)  **P-4**  2 walking sessions (2x30 min)  **P-6**  1 walking session (42 min)  **P-12**  3 walking sessions (45 and 90x2 min)  1 session at the gym (50 min)  **P-13**  2 walking sessions (45 and 30 min)  1 jogging session (30 min) |
| 2 | Walking (P-1, 2, 4, 5, 6, 7, 8, 9, 10 and 13)  ST (P-6, 8 and 10)  Cycling (P-2 and 8)  Activities at the gym (P-12)  Table tennis (P-1)  Cross-country skiing (P-2)  Gardening (P-5)  Football (P-6)  **Meeting guidelines (P-1, 2, 9)** | **P-1***  1 table tennis session (93 min)  **P-2***  4 cross-country skiing sessions (50x3 and 30 min)  **P-8**  2 cycling session (2x25min)  **P-9***  2 walking sessions (60 and 120 min)  **P-10**  1 ST session (45 min) | **P-1***  2 table tennis sessions (24 and 53 min)  **P-2***  1 walking session (54 min)  1cycling session (30 min)  **P-6**  1 walking session (76 min)  1 ST session (20 min)  1session of football (30 min)  **P-9***  4 walking sessions (20, 25 and 2x40 min)  **P-10**  2 ST sessions (2x20 min)  **P-12**  1 session at the gym (90 min) | | **P-1**  5 walking sessions (10, 2x15 and 2x60 min)  **P-2**  9 walking sessions (7x15 and 3x20 min)  **P-4**  3 walking sessions (2x15 and 30 min)  **P-5**  1 walking session (45 min)  **P-6**  2 walking sessions (20 and 25 min)  **P-7**  7 walking sessions (7x20min)  **P-8**  3 walking sessions (2x25 and 30 min)  1 ST session (15 min)  **P-10**  3 walking sessions (2x45 and 80 min)  **P-13**  3 walking sessions (2x50 and 95 min) |
| 3 | None activities (P-4 and 13)  Walking (P-1, 2, 5, 7, 9, 10 and 12)  ST (P-6, 8 and 10)  Jogging (P-8, 10 and 12)  Activities at the gym (P-12)  Table-tennis (P-1 and 9)  Cross-country skiing (P-2)  Gardening (P-8 and 10)  **Meeting guidelines (P-2, 10, 12)** | **P-2***  1 cross-country skiing session (60 min)  **P-6**  1 ST session (20 min)  **P-8**  1 running session (30 min)  **P-9**  1 table tennis session (30 min)  **P-10***  1 jogging session (30 min)  2 ST sessions (25 and 30 min.)  **P-12***  2 jogging sessions (75 and 90 min.) | **P-1**  2 table tennis sessions (33 and 44 min)  **P-2***  2 cross-country skiing sessions (32x2 min)  **P-9**  2 walking sessions (2x20 min)  **P-10***  1 ST session (30 min)  **P-12***  1 walking session (70 min)  1 jogging session (90 min.)  1 session at the gym (80 min.) | | **P-1**  1 walking session (15 min)  **P-2**  11 walking sessions (8x15, 2x30 and 40 min)  **P-7**  10 walking sessions (10x20min)  **P-8**  1 ST session (30 min)  1 session gardening (60 min)  **P-10**  1 walking sessions (50 min)  1 Gardening session (20min.)  **P-12**  1 walking session (68 min.) |
| **BEP 2** | **Type of activities** | **High intensity session**  **(> 15 Borg scale)** | **Moderate intensity sessions**  **(12-14 Borg scale)** | | **Low intensity sessions**  **(< 11 Borg scale)** |
| **4** | None activities (P-1, 5, 13)  Walking (P-2, 4, 6, 7, 8, 9, 10, 12)  ST (P-10)  Jogging (P-2)  Activities at the gym (P-12)  Cross-country skiing (P-2)  **Meeting guidelines (P-2, 9)** | **P-2***  1 jogging session (40 min)  2 cross-country skiing sessions (48 and 95 min)  **P-9***  2 walking sessions (2x160 min)  **P-10**  1 session ST (20 min) | **P-2***  1 walking session (40 min)  **P-12**  1 session at the gym (75 min) | | **P-2**  5 walking sessions (3x15, 30 and 40 min)  **P-4**  2 walking sessions (15 and 240)  **P-6**  7 walking sessions (4x10, 20 and 2x50 min)  **P-7**  4 walking session (4x45min)  **P-10**  3 walking sessions (20, 40 and 60 min.)  **P-12**  2 walking sessions (2x40 min.) |
| **5** | None activities (P-6, 8 )  Walking (P-1, 2, 4, 7, 9, 10, 12, 13)  ST (P-12)  Activities at the gym (P-12)  Cross-country skiing (P-2)  Gardening (P-5 and 12)  Curling (P-1)  Swimming (P-10)  Housekeeping (P-10)  Hiking (P-10)  **Meeting guidelines (P-1, 9, 10)** | **P-2**  1 cross-country skiing session (44 min)  **P-9***  3 walking sessions (10, 30 and 60 min) | **P-1***  1 session curling (185 min)  **P-10***  1 session hiking (360 min)  1 session housekeeping (45 min)  **P-12**  1 ST session (84 min)  **P-13**  1 walking session (30 min) | | **P-1**  1 walking sessions (60 min)  **P-2**  7 walking sessions (2x10, 15, 2x20, 40 and 50 min.)  **P-4**  1 walking session (25 min)  **P-7**  3 walking sessions (3x45)  **P-10**  1 walking session (10 min)  1 session swimming (30 min)  1 session housekeeping (30 min)  **P-12**  2 walking sessions (30 and 45 min)  1session at the gym (60 min)  1 session gardening (60 min) |
| **6** | None activities (P-1, 6, 8 )  Walking (P-2, 4, 5, 7, 9, 10, 13)  Activities at the gym (P-12)  Cross-country skiing (P-2)  Gardening (P-4 and 5)  Housekeeping (P-4)  Paddling (P-10)  Football (P-10)  Cross-fit (P-10)  **Meeting guidelines (P-10, 12)** | **P-2**  1 cross-country skiing session (58 min)  **P-9**  2 walking sessions (15 and 10 min)  **P-10***  1 cross-fit session (30 min) | **P-10***  2 paddling sessions (60 and 270 min)  1 football session (30 min)  **P-12***  2 sessions at the gym (85 and 90 min)  **P-13**  1 walking session (30min) | | **P-2**  21 walking sessions (21x20 min)  **P-4**  3 walking sessions (20 and 2x40 min)  1 session gardening (240 min)  1 session with housekeeping (30 min)  **P-5**  1 walking session (20 min)  1 session gardening (> 240 min)  **P-10**  1 walking session (90 min)  **P-12**  2 sessions at the gym (45 and 90 min) |
| **BEP 3** | **Type of activities** | **High intensity session**  **(> 15 Borg scale)** | **Moderate intensity sessions**  **(12-14 Borg scale)** | **Low intensity sessions**  **(< 11 Borg scale)** | |
| **7** | None activities (P-1, 5, 6, 8, 13).  Walking (P-2, 4, 7, 9, 10, 12)  Running (P-2)  Gardening (P-4)  **Meeting guidelines (P-9)** | **P-2**  1 running session (44 min)  **P-9***  3 walking sessions (180, 30 and 40 min) | None | **P-2**  4 walking sessions (10, 15, 30 and 45 min)  **P-4**  2 walking sessions (20 and 45 min)  1 session gardening (30 min)  **P-10**  2 walking sessions (15 and 30 min)  **P-12**  1 walking session (30 min) | |
| **8** | None activities (P-1, 2, 4, 6, 8, 9, 13)  Walking (P-5, 7, 10)  ST (P-10)  Activities at the gym (P-12)  Gardening (P-5, 12)  **Meeting guidelines (P-10)** | **P-10***  1 ST session (30 min) | **P-10***  2 walking sessions (45 and 160 min)  1 ST session (40 min)  **P-12**  1 session at the gym (80 min) | **P-12**  1 session at the gym (90 min)  1 session gardening (60 min) | |
| **9** | None activities (P-1, 2, 6, 8, 9, 13)  Walking (P-4, 5, 7)  ST (P-10)  Activities at the gym (P-12)  Gardening (P-5)  **Meeting guidelines (P-12)** | **P-10**  1 ST session (20 min) | **P-4**  3 walking sessions (40 and 20x2 min)  **P-12***  3 sessions at the gym (2x90min) | **P-5**  2 sessions of gardening (> 120 min) | |

ST- strength training
